# Formation of the 42-mer Amyloid **β** Radical and the Therapeutic Role of Superoxide Dismutase in Alzheimer's Disease

**DOI:** 10.4061/2011/654207

**Published:** 2011-01-16

**Authors:** Kazuma Murakami, Takahiko Shimizu, Kazuhiro Irie

**Affiliations:** ^1^Laboratory of Organic Chemistry in Life Science, Division of Food Science and Biotechnology, Graduate School of Agriculture, Kyoto University, Sakyo-ku, Kyoto 606-8502, Japan; ^2^Molecular Gerontology, Tokyo Metropolitan Institute of Gerontology, Itabashi-ku, Tokyo 173-0015, Japan

## Abstract

Oxidative stress is closely involved in age-related diseases and ageing itself. There is evidence of the leading contribution of oxidative damage to neurodegenerative disease, in contrast to other diseases where oxidative stress plays a secondary role. The 42-mer amyloid *β* (A*β*42) peptide is thought to be a culprit in the pathogenesis of Alzheimer's disease (AD). A*β*42 aggregates form the oligomeric assembly and show neurotoxicity, causing synaptic dysfunction. A*β*42 also induces tissue oxidation (DNA/RNA, proteins, and lipids) through trace metals (Cu, Zn, and Fe), which can be protected by antioxidant enzymes, vitamin C, and vitamin E. Superoxide dismutase catalyzes the conversion of toxic superoxide radical to less reactive hydrogen peroxide, contributing to protection from AD. Here we review the involvement of oxidative stress in AD progression induced from an imbalance between the radical formation of A*β*42 *itself* together with unique turn structure at positions Glu22 and Asp23 and several defense systems.

## 1. Oxidative Stress in Ageing—The Involvement of Superoxide Radical

**Figure 1:**
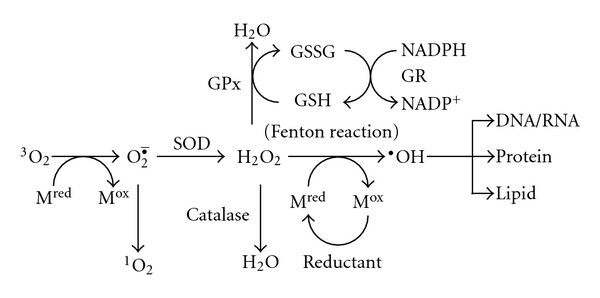
Generation of reactive oxygen species and defense systems in the cell. M^red^ or M^ox^, reduced or oxidized form of metals; SOD, superoxide dismutase; GSH, reduced glutathione; GSSG, oxidized glutathione; GR, glutathione reductase; GPx, glutathione peroxidase; NADPH or NADP^+^, reduced or oxidized nicotinamide adenine dinucleotide phosphate; VC, vitamin C; VE, vitamin E.

Oxidative stress caused by reactive oxygen species (ROS) has been implicated in numerous age-related diseases and ageing itself [1, 2]. ROS include superoxide anions, hydrogen peroxide, hydroxyl radicals, and singlet oxygen. ROS are also involved in neurodegeneration such as Alzheimer's disease (AD), Parkinson's disease, and amyotrophic lateral sclerosis, because the brain is one of the most vulnerable tissues in the body to oxidative injuries based on its high rate of oxygen consumption [3]. The hydroxyl radical is believed to be one of the main stimuli of oxidative damage (Figure 1) and reacts with several biomolecules, leading to the formation of 8-hydroxydeoxyguanosine (8-OHdG)/8-hydroxyguanosine (8-OHG) in DNA/RNA, the formation of methionine sulfoxide, carbonylation in proteins, and lipid peroxidation. In particular, lipid peroxidation can lead to the production of 4-hydroxyl nonenal (4-HNE), malondialdehyde (MDA), and thiobarbituric acid-reacting substances (TBARS) as byproducts. The subsequent processes to hydroxyl radical could be involved in peroxynitrite formation by stimulating inducible nitric oxide synthase (i-NOS). Hydrogen peroxide (H_2_O_2_) is less reactive but is involved in the Fenton reaction (Haber-Weiss procedure, Figure 1), providing the hydroxyl radical.

On the other hand, the superoxide radical is also biologically toxic, especially under the condition of radical-radical reactions which could occur at a diffusion-controlled rate. It presents widely in high quantity. A small fraction (0.4%–4%) of oxygen utilized in the mitochondria is reduced by single electron transfer during the initial step of the electron transport chain, followed by the generation of superoxide radical [4]. Catalase and peroxidases (such as glutathione peroxidase: GPx), which exists in ubiquitous tissues, can eliminate H_2_O_2_ generated from superoxide radicals (Figure 1).

Superoxide dismutases (SODs) are the main antioxidant enzymes that convert superoxide anions to H_2_O_2_, protecting cells and tissues from ROS generated from endogenous and exogenous sources [5]. SODs consist of three types of isoforms expressed in mammalian cells: copper/zinc SOD (CuZn-SOD, SOD1), which is located in the cytoplasm, manganese SOD (Mn-SOD, SOD2), which exists in the mitochondrial matrix, and extracellular SOD (EC-SOD, SOD3), which is also a complex of CuZn. Some CuZn-SOD is also seen in the intermembrane space of mitochondria [6].

Van Remmen is one of the most influential leaders in ageing research. She and her colleagues have reported several ageing symptoms of hepatic carcinoma [7] and muscle atrophy [8] in CuZn-SOD knockout mice. They also studied the physiological role of several antioxidant enzymes in longevity using gene-disrupting mice [9]. Notably, CuZn-SOD-deficient mice showed the multiple pathologies in several tissues with decreased lifespan compared to wild-type mice. Fujii and colleagues also indicated that hemolytic anemia was triggered by autoantibody production in CuZn-SOD-deficient mice [10].

Our group has reported that CuZn-SOD-deficient mice showed skin thinning [11] as well as increased drusen formation, which is a typical characteristic of age-related macular degeneration as neurodegeneration [12] and fatty liver [13]. Taken together, these observations demonstrate that CuZn-SOD knockout mice have the potential to be a valuable animal model for investigating human ageing. On the other hand, Shimizu and colleagues generated various tissue-specific Mn-SOD conditional knockout mice using a Cre-loxp system because total knockout of Mn-SOD induces neonatal lethality in mice [14, 15], liver-specific Mn-SOD knockout mice which show no obvious morphological abnormalities or biochemical changes in the liver [16], heart/muscle-specific Mn-SOD-deficient mice which exhibit dilated cardiomyopathy with the downregulation of specific biomolecules in the mitochondria [17], and skeletal muscle-specific Mn-SOD knockout mice which develop severe disturbance of exercise activity without muscle atrophy [18]. Furthermore, they found severe phenotypes in the brains of brain-specific Mn-SOD-deficient mice showing a spongiform encephalopathy-like pathology associated with gliosis [19]. The most abundant ROS within cells influencing synaptic plasticity, memory function, and neuronal death is considered to be the superoxide radical [20]; this suggests that SOD plays a protective role in neurodegeneration. We introduce the relevance of oxidative stress to AD in the following section.

## 2. A*β* Theory in Alzheimer's Disease

AD is generally characterized by the aggregation of amyloid *β* (A*β*) in senile plaques. A*β* mainly consists of 40- and 42-residue amyloid *β* peptides (A*β*40, A*β*42), secreted from amyloid precursor protein (APP) by two proteases (*β*- and *γ*-secretases) [21, 22]. A*β*42 plays a more critical role in the pathogenesis of AD than A*β*40 because A*β*42 aggregates more extensively to form fibrils and shows stronger neurotoxicity [23]. On the other hand, there is increasing evidence that the oligomeric assembly of A*β* could induce memory decline and synaptotoxicity in AD [24], while mature plaques were reported to be nontoxic [25, 26] and to serve as a store of the toxic assembly of A*β* [27].

Studies on several kinds of A*β* oligomer associated with neurotoxicity or synaptotoxicity have been accumulated. Recently, Teplow and colleagues summarized and overviewed A*β* assembly [28]: paranucleus, protofibrils (24–700 mer) [29], A*β*-derived diffusible ligands (ADDL, ~53 kDa) [30], Aβ*56 (~56 kDa, 12-mer) [31], amylospheroid (~150–700 kDa) [32], A*β*O (~90 kDa, 15–20 mer), annulus (150–250 kDa), and *β*amyball. Selkoe and colleagues suggested that A*β* dimers are the smallest synaptotoxic species and that plaque cores are largely inactive but sequester or release dimers [33]. They developed unique oligomer specific-ELISA using 82E1 antibody, whose epitope is N-terminal, for both antigen capture and detection, to reveal a clear correlation of the oligomer levels in the plasma and brain extracts with various cognitive levels of AD patients [34]. Oligomeric molecules of A*β* are believed to consist of 2 or 3x *n*-multimers based on the dimer or trimer, respectively. To elucidate the mechanism of A*β* oligomerization, many scientists have developed a method or detection tools. Bitan and colleagues created a method of the photoinduced cross-linking of unmodified proteins to prepare the oligomers in large quantity [35]. Glabe and colleagues generated a conformation-dependent antibody (A11 clone) against A*β* oligomers, which does not recognize fibrils and also reacts with other types of amyloid oligomers, such as *α*-synuclein in Parkinson's disease, polyglutamine in Huntington's disease, and prion peptide 106–126 in prion disease [36]. Recently, they reported the fibril-specific, conformation-dependent antibody (OC clone), recognizing soluble oligomers ranging from a dimer to greater than 250 kDa [37].

## 3. Role of Trace Metals and Formation of A*β* Radical in Alzheimer's Disease

In 1965, Terry and Pena. first reported the relevance of aluminum to the pathology of AD; they injected aluminum salts into the rabbit brain, resulting in neurofibrillary tangle formation [38], which is another hallmark of AD. Although aluminum in the diet or drinking water had been long believed as a risk factor for AD [39], Ehmann et al. in 1986 showed that this hypothesis for AD was an artifact [40].

It is known that transition metals, such as Cu, Zn, and Fe, are enriched in senile plaques [41]. A*β* causes protein oxidation, DNA/RNA oxidation, and lipid peroxidation* in vitro* and *in vivo*, possibly by aggregating to generate radicals *via* a trace of metal ions (Cu and Zn) [41–44]. The imbalance of copper homeostasis is also implicated in the etiology of AD [45]. The neurotoxic effects of A*β*42 and A*β*40 in cell culture correlate with the ability to reduce Cu(II) to Cu(I) and to generate H_2_O_2_ in a cell-free system [46]. The direct interaction of metals with A*β* in the N-terminal region is essential for its aggregation and neurotoxicity. In complex formation with Cu(II) [47, 48], each of the three histidine residues at positions 6, 13, and 14 of A*β*42, Tyr10 [46, 49–52], and Asp1/Asp7 [53] may be involved. Recent ESR studies by Drew et al. suggested that the Ala2 carbonyl could be involved in the Cu(II) coordination [54]. Tyr10 is easily oxidized to the tyrosyl radical by Cu(II), leading to the production of H_2_O_2_ [55]. Quite recently, Ono et al. reported that UK (H6R) and Tottori (D7N) mutations in the N-terminal regions accelerated the ability to form oligomers and enhanced cytotoxicity [56]. These mutations might change the binding mode of metal with A*β* peptides, resulting in the increased ability to form toxic oligomers.

Based on the metal etiology in AD, therapeutics using metal chelators might be promising to prevent plaque formation by extracting the metals. Bush and colleagues treated an APP transgenic mouse with a CuZn chelator, clioquinol (8-hydroxy quinoline), showing the effective removal of plaque depositions [57]; however, it might alter the homeostasis of copper and counteract the intracellular copper-depleting effects of APP in initial clinical trials of the treatment of AD [58]. Eventually, it was removed from the market by FDA due to difficulties associated with chelation of Co(II) involved in vitamin B12. They also mentioned that the problems were also due to the large magnitude difference in affinity of Cu(II) between clioquinol and A*β*42, in which *K*_*d*_ of Cu(II) for clioquinol and A*β*42 are nanomolar and attomolar, respectively [59]. Other processes for plaque removal by clioquinol could be involved. Recently, they advanced the chelating strategy into the second-generation clioquinol analogue, PBT2, which outperformed clioquinol by markedly decreasing soluble interstitial A*β* and rescuing cognitive impairment [60]. PBT2 was already found to reverse frontal lobe functional deficits and to decrease A*β*42 in a phase IIa clinical trial [61].

Butterfield and colleagues pioneered the contribution of Met35 to the neurotoxicity and oxidative effects of A*β* [44, 62]. The oxidized form of Met35 was detected both in the brains of AD patients [63] and the APP transgenic mouse model [64]. They suggested the reactive form of Met35 in A*β*42 as an *S*-oxidized radical cation, abstracting an allylic hydrogen of phospholipid acyl chains to give allyl radicals, followed by lipid peroxidation [43]. The methionine sulfoxide reductase is known to reverse methionine oxidation. Moskovitz and colleagues reported that a knockout mouse of one isoform of this enzyme caused enhanced neurodegeneration in the brain hippocampus, implying that the oxidation of Met residue plays a role in brain pathology [65].

**Figure 2:**
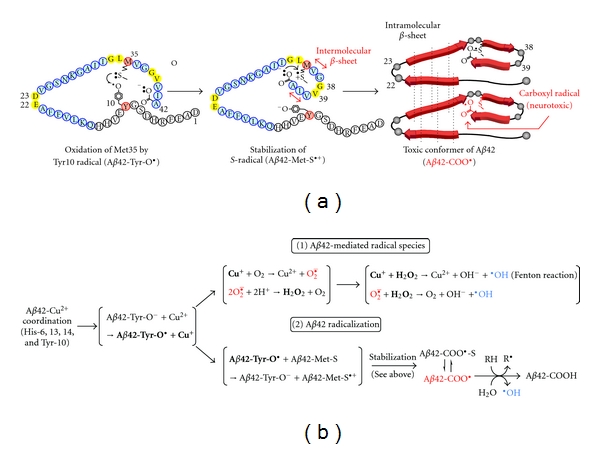
(a) Proposed mechanism of formation and stabilization of A*β*42 radical for long-lasting oxidative stress in AD and the toxic conformer of A*β*42 [73]. (b) Scheme of generation of A*β*42-mediated radical species (superoxide radical and hydroxyl radical) and the long-lasting A*β*42 radical in the pathogenesis of AD.

The S-oxidized radical cation in Met35 is generally too unstable to cause oxidative damage continuously [66]. A stabilization mechanism for long-lasting oxidative stress in AD progression is required. We have proposed the emerging role of the turn formation at Glu22 and Asp23 in the pathogenesis of AD [67, 68] and its contribution to oligomer formation [69] following intracellular amyloidgenesis [70]. Our continuous studies using a systematic proline replacement, solid-state NMR, and ESR have elucidated A*β*42-mediated neurotoxicity *in vitro*; the central turn formation could bring Tyr10 radical generated through trace metals accompanied by the generation of H_2_O_2_, which are moved close to Met35, resulting in the production of the S-oxidized radical cation (Figure 2(a)). The systematic proline replacement of A*β*42 proposed that not only the turn formation at Glu22 and Asp23 but the turn at Gly38 and Val39 increases aggregation and neurotoxicity [71]. This additional C-terminal turn could enable the carboxylate anion at Ala42 to interact with the S-oxidized radical cation by forming S-*O* bonding through an intramolecular *β*-sheet at positions 35–37 and 40–42 (Figure 2(a)). The resultant hydrophobic core in the C-terminus would enhance A*β*42 aggregation, sequestering or releasing the radical species for long-lasting oxidative stress in AD. If considered for the lower toxicity of A*β*40 toxicity, the *S*-oxidized radical cation of Met35 might not be fully stabilized by the incomplete association of Met35 radical with the carboxylate anion at Val40 (Figure 2(a)) or by the labile electrostatic interaction between the sulfur atom of Met35 and the amide carbonyl group of Ile31 under the condition of *α*-helix formation in the C-terminal region [72]. Collectively, the formation of toxic A*β* radicals generated through trace metals could induce the malfunction of signal transduction pathways after the interaction with membranes. This mechanism (Figure 2(a)) can in part explain why A*β*42 is more neurotoxic than A*β*40 [73]. The following generation of superoxide radical and hydroxyl radical occasionally accompanied with the stabilization of A*β*42 radical could attack the membranes and other macromolecules (Figure 2(b)). At least two A*β*42-mediated pathways are assumed.

Recently, Butterfield and colleagues advanced the Met35 theory into *in vivo* analysis using APP transgenic mice with V717F (Indiana) and M631L mutations corresponding to the substitution of Met35 with Leu in the A*β* sequence [74], which showed the prevention of oxidative damages in tissues and senile plaque in the brain. Unexpectedly, M35L mutation in mice exhibited almost no effects on memory and learning impairments in the Morris water maze [74], indicating that oxidative stress may be neither required nor sufficient for memory loss. Quite recently, Bitan and colleagues suggested that Met35 is not necessary for A*β* toxicity despite its significant role in aggregation [75]. Other mechanisms in addition to Met35 will occur for the complete explanation of A*β*42-induced neurotoxicity.

A relationship between A*β* oligomers and oxidative stress has been noted; Klein and colleagues proposed that ADDL induce long-term potentiation associated with oxidative damage *in vitro* [76]. Barnham and colleagues proposed that A*β* generated dityrosine cross-linked dimers through oxidation of the phenolic hydroxyl group at Tyr10 under oxidative conditions [55], and that generic dityrosine levels were increased in the AD brain [77]; however, it is unclear whether A*β*-mediated oxidative damage observed *in vitro* is relevant to *in vivo* disease.

## 4. Oxidative Stress and Antioxidants in Alzheimer's Disease

There is increasing evidence that oxidative stress is a prominent and early feature of AD [78]. The Fenton reaction mediated by iron or copper can result in the oxidative damage of nucleic acids. Smith and colleagues proposed that 8-OHdG is an established marker of nuclear DNA oxidation for AD pathology [78]. Butterfield and colleagues proposed that HNE is produced by A*β*-induced lipid peroxidation [79]. Redox proteomics using human AD brains showed that the elevation of TBARS was associated with the numbers of neuritic but not diffuse plaques [80].

On the other hand, glutathione (GSH), a tripeptide, is biosynthesized in the cytoplasm and normally exists in the mitochondrial matrix as a reduced form [81] because glutathione reductase (GR) plays a role in maintaining the ratio of GSH to GSSG through the oxidation of nicotinamide adenine dinucleotide phosphate (NADPH) (Figure 1). GSH maintains the integrity of the plasma membrane and adenosine triphosphate (ATP) in the synaptosomes as an antioxidant. Under severe oxidative stress, the accumulation of GSSG occurs together with protein modification. Studies using AD brains by Balaz and Leon revealed almost no changes in the levels of glutathione and catalase [82], which is an important enzyme to convert H_2_O_2_ into H_2_O and O_2_ (Figure 1). In contrast, Gsell et al. reported the decreased activity of catalase in AD brains [83]. Some markers of oxidative stress will be vulnerable or not to the formation of A*β* radical in AD pathology. Alternatively, the technical artifact during the isolation of proteins may affect the results for oxidative levels among different research groups.

Table 1 summarizes *in vivo* studies on the involvement of these enzymes in AD. The cytochrome *c* oxidase (COX) is involved in respiratory electron transport in the mitochondrial inner membrane. There are several studies on the correlation of the reduced activity of COX and increased oxidative stress in AD brains [93, 94]. Fukui et al. crossed an AD transgenic mouse with a neuron-specific *COX10 *knockout mouse and reported that COX deficiency failed to increase both senile plaques and oxidative damage in AD progression in contrast to their expectations [90]. NADPH oxidase, believed to be one of the major ROS sources in the brain, participates in the generation of superoxide radical by transferring electrons across the membrane into molecular oxygen [95]. Genetic inactivation of *Nox2*, an isozyme of the catalytic subunit of NADPH oxidase, prevented oxidative stress, A*β*-derived neurovascular dysfunction, and behavioral impairment without affecting A*β* assembly [91]. Binding of the transcription factor nuclear factor E2-related factor 2 (Nrf2) to the antioxidant response element (ARE) enhancer sequence is known to induce the endogenous defense system against oxidative stress. The Nrf2-ARE pathway is activated in response to ROS, triggering the expression of antioxidant enzymes. Kanninen et al. reported that intrahippocampal injection of Nrf2 mitigated the spatial impairment of AD mice (APP/PS1 mice) associated with increased plaque formation and heme oxygenase-1 levels [96].

Glutathione peroxidase (GPx) is also a key modulator in the neuronal system, participating in the elimination of H_2_O_2_. Overexpression of GPx4, an isoform expressed in the membrane, reduced the lipid peroxidation of mice after exposure to diquat, known as a herbicide, and induced mice resistant to apoptosis from oxidants [97]. Embryonic fibroblasts of catalase transgenic mice are more resistant to toxic H_2_O_2_ [98]. Thioredoxin plays a role in repairing the oxidation of cysteine residues in proteins [99]. Yodoi and colleagues generated transgenic mice overexpressing human thioredoxin, which reduced oxidative stress and extended its lifespan [100]. The therapeutic effects of these antioxidative enzymes against AD are expected although no studies on their role in AD pathology have been reported.

## 5. Therapeutic Role of Superoxide Dismutase in Alzheimer's Disease

The role of SOD in AD pathogenesis has long been controversial. Several studies have shown decreased SOD in the frontal cortex of AD patients [101] whereas a slight elevation of SOD was documented in the caudate nucleus of AD patients [102]. Alternatively, other researchers have suggested that almost no changes in SOD levels are found in AD brains [83]. Quite recently, Ansari and Scheff reported a strong correlation between several oxidative damage levels using various dementia subjects with negligible levels of premortem hypoxia in order to eliminate the possibility of affecting protein integrity [103]. As shown in Table 1, Melov et al. suggested that mitochondrial oxidative stress could induce the hyperphosphorylation of tau at Ser396 using Tg2576 transgenic AD mouse model [85]. There have also been reports on the role of Mn-SOD in AD pathology; AD transgenic mouse models crossed with *Sod2^+/−^* resulted in increased accelerated behavioral deficits [86] or senile plaques [87] (Table 1). Quite recently, our group proposed the involvement of CuZn-SOD in AD progression; the superoxide radical in the cytoplasma induced A*β* oligomerization and early cognitive impairment in Tg2576, and these phenomena notably preceded oxidative damage (Murakami, K. et al., submitted) (Table 1). Our findings do not contradict the implication by Marlatt et al. that oxidative damage occurs primarily within the cytoplasm rather than the mitochondria [3].

In the therapy of AD by SODs, cerebral endothelial dysfunction in the AD mouse model can be improved by overexpression of *Sod1* [84] (Table 1) or the administration of SOD [104]. Bayer et al. proposed that dietary intake of Cu stabilizes CuZn-SOD activity and decreases A*β* production in the APP transgenic mouse model [105]. On the other hand, overexpression of *Sod2* rescued several markers for oxidative stress associated with AD-like pathologies in two representative lines of AD model mice (Tg2576 [88] and Tg19959 [89]) (Table 1). Under the excessive reduced redox-acitve metal ions, the adverse effects due to hydroxyl radical formation should be taken into account. The therapeutic treatment of both SOD and catalase mimetics (e.g., EUK-8 [106]) could be one of promising approaches.

Breteler and colleagues performed a clinical survey of the dietary intake of antioxidants and the risk of AD based on over 5,000 participants in the Netherlands [107]. It was suggested that high dietary intake of vitamin C and vitamin E might lower the risk of AD. Dementia control by vitamin C and vitamin E has long been discussed [108–112]. Interestingly, Rinaldi et al. suggested the correlation of vitamin C and SOD levels with the dementia status [113]. SOD might be one of the most vulnerable indicators as an antioxidant enzyme in AD and cognitive dementia. Alternatively, it was reported that environmental enrichment prevented AD-like pathology associated with elevated CuZn-SOD and Mn-SOD levels [114].

## 6. Conclusions

One of the most accepted knowledge in the etiology of AD is thought to be the free-radical theory; however, it remains to be determined whether oxidative stress is a cause or effect in AD. A*β*42 aggregates (oligomerizes) induce neurotoxins by interacting with trace metals at Tyr10 or in the N-terminal region, leading to tissue oxidation by an *S*-oxidized radical cation in Met35. There are three means of defense from A*β*42-dependent AD pathology: (1) to slow the rate of A*β*42 aggregation, (2) to decrease the production of A*β*42 by downregulating the activity of *β*- or *γ*-secretase, and (3) to enhance protease activity (such as neprilysin [115], an insulin-degrading enzyme [116]) against A*β*42. Oxidative stress may affect one or all of the protective mechanisms. Antioxidant enzymes including SOD or dietary supplements of vitamin C and vitamin E could counteract these dysfunctions. Food treatments for prevention are a better choice to maintain the quality of life. There are increasing reports on the inhibitory effects of natural products such as several flavonoids [117], vitamin A [118], and vitamin E [119] on AD pathology *in vivo*. Nishida et al. reported *α*-tocophenol transfer protein-knockout mice, in which A*β* deposits accumulated by decreasing the clearance of A*β* peptide from the brain and blood [92] (Table 1). Quite recently, we discovered the potential of silymarin [120], the active ingredient of milk thistle extract which is long used as a hepatoprotective medicine, and vitamin C (Murakami, K. et al., submitted) for AD prevention. Further research on structural analysis of the inhibitory mechanism is under investigation to effectively develop inhibitors with few adverse effects.

## Figures and Tables

**Table 1:** Studies on involvement of oxidative stress in AD *in vivo. *

Objected mice	APP mice	Behavior	A*β*-dependent pathology	ROS marker	References
CuZn-SOD KO	Tg2576	Early memory loss	Oligomer↑, P-tau↑	8-OHdG↑, Protein carbonyls↑	Submitted
CuZn-SOD Tg^a^	Tg1130H	NT	Cerebral dysfunction↓	NT	[84]^a^
Mn-SOD hetero KO	Tg2576^b^, J20^c^, Tg19959^d^	Early memory loss^c^	A*β* depositions↑^c,d^, P-tau↑^b^,	NT	[85]^b^, [86]^c^, [87]^d^
Mn-SOD Tg	Tg2576^e^, Tg19959^f^	Improved memory loss^e,f^	A*β*42/ A*β*40↓^e^, Plaque↓^f^,	DHE↓^e^, Catalase↑^f^, Protein carbonyls↓^f^,	[88]^e^, [89]^f^
COX10 KO	APPswe/PSEN1ΔE9	NT	Plaque↓, A*β*42↓	8-OHdG↓, Protein carbonyls↓	[90]
Nox2 KO	Tg2576	Improved abnormal behavior	Unchanged (A*β*42, A*β*42, plaque)	DHE↓	[91]
*α*-tocophenol transfer protein KO	Tg2576	NT	A*β*40↑, IDE↓	Unchanged	[92]

Abbreviations: A*β*, amyloid *β*; AD, Alzheimer's disease; APP, amyloid precursor protein; COX, cytochrome *c* oxidase; DHE, dihydroethidium; IDE, insulin-degrading enzyme; KO, knock out; Nox, NADPH oxidase; NT, not tested; 8-OHdG, 8-hydroxydeoxyguanosine; PSEN, presenilin; P-tau, phosphorylated tau; ROS, reactive oxygen species; SOD, superoxide dismutase; Tg, transgenic; ↑, increased; ↓, decreased.
